# Partial Discharge Fault Diagnosis Based on Multi-Scale Dispersion Entropy and a Hypersphere Multiclass Support Vector Machine

**DOI:** 10.3390/e21010081

**Published:** 2019-01-17

**Authors:** Haikun Shang, Feng Li, Yingjie Wu

**Affiliations:** 1 College of Electrical Engineering, Northeast Electric Power University, Jilin 132012, China; 2 State Grid Electric Power Research Institute, Xinjiang 830011, China; 3 College of Automation Engineering, Northeast Electric Power University, Jilin 132012, China

**Keywords:** PD, fault diagnosis, variational mode decomposition, multi-scale dispersion entropy, HMSVM

## Abstract

Partial discharge (PD) fault analysis is an important tool for insulation condition diagnosis of electrical equipment. In order to conquer the limitations of traditional PD fault diagnosis, a novel feature extraction approach based on variational mode decomposition (VMD) and multi-scale dispersion entropy (MDE) is proposed. Besides, a hypersphere multiclass support vector machine (HMSVM) is used for PD pattern recognition with extracted PD features. Firstly, the original PD signal is decomposed with VMD to obtain intrinsic mode functions (IMFs). Secondly proper IMFs are selected according to central frequency observation and MDE values in each IMF are calculated. And then principal component analysis (PCA) is introduced to extract effective principle components in MDE. Finally, the extracted principle factors are used as PD features and sent to HMSVM classifier. Experiment results demonstrate that, PD feature extraction method based on VMD-MDE can extract effective characteristic parameters that representing dominant PD features. Recognition results verify the effectiveness and superiority of the proposed PD fault diagnosis method.

## 1. Introduction

Partial discharge (PD) is an important symptom of insulation degradation for electrical equipment. PD fault diagnosis plays an irreplaceable role in the evaluation of insulation condition [1]. PD feature extraction is an important step in insulation fault diagnosis. The common methods include statistical atlas (SA) [2], wave analysis (WA) [3] and wavelet transform (WT) [4]. However, SA has the limitations of high request of sampling rate, large data size and slow speed of data processing which are not suitable for on-line monitoring. Besides, it is difficult to extract PD phase information during statistical atlas construction. WA is easily influenced by electromagnetic interference. WT has some inherent limitations such as the difficulty of selection of the wavelet basis, wavelet thresholds, decomposition levels, and so on [5].

Empirical mode decomposition (EMD), as an adaptive signal processing method that decomposes a time series into some limited intrinsic mode functions (IMFs). It is widely used in the areas of fault detection, signal processing and data compression [6,7,8]. However, due to the problems of ending effects and mode mixing in non-stationary signal decomposition, EMD is limited in practical applications. Variational mode decomposition (VMD) is a new signal decomposition method, which is widely applied in electrical fault feature extraction [9]. It is a non-recursive variational decomposition model. In VMD, the central frequency and bandwidth of each mode are determined by searching the optimal solution of the variation model. VMD can solve the problems of mode mixing and ending effects in traditional EMD methods [10]. In this paper, VMD is employed for PD signal decomposition to extract effective IMFs from PD signals.

In order to quantify the PD feature information extracted by VMD, entropy theory is introduced. Entropy, as a measure of uncertainty or irregularity, was widely applied in fault diagnosis recently [11]. It was first introduced by Shannon in 1948 [12]. Afterwards, approximate entropy (AE) was put forward by Pincus [13], which provided one dimensionless index representing signal features. It was suitable for both deterministic and random signals. However, AE is heavily relied on the data length. Moreover, its estimated value is uniformly lower than expected ones when processing the short dataset [14]. To overcome the weakness of AE, Richman and Moorman proposed sample entropy (SE) [15]. Due to the insensitivity to the data length and immunity to the noise in data, SE has attracted a great deal of attention. However, SE is not fast enough for some real-time applications, especially for long signals [16]. Another widely used regularity indicator is permutation entropy (PE), which is based on the order relations among values of a signal [17]. Although PE is conceptually simple and computationally fast, the method does not consider the mean value of amplitudes and differences between amplitude values [18]. In this paper, a new irregularity indicator is introduced, named dispersion entropy (DE) [19]. The method tackles the abovementioned PE and SE limitations [20]. Because of the relevance and the possible usefulness of DE in several signal analyses, it is important to understand the behavior of the technique for various kinds of classical signal concepts such as amplitude, frequency, noise power, and signal band-width. However, DE estimates the complexity at a single scale [21], which gives rise to unacceptable result when applied to analyze the multiple time scales data [22]. Regarding this disadvantage, a multi-scale dispersion entropy (MDE) procedure was put forward to estimate the complexity of the original time series over a range of scales [23]. In this work, MDE is employed to quantify the PD feature information.

In recent years, a great number of intelligent algorithms have been used in PD fault diagnosis. Support vector machine (SVM) [24], as a learning machine based on kernel functions, that has the property of global optimization and strong generalization ability. However, using hyperplane recognition model, SVM can’t accurately classify the samples with nonuniform state distribution. In addition, SVM is restricted in practical application for its inherent binary classification properties [25].

Hypersphere Support Vector Machine (HSSVM), based on SVM, was first proposed by Scholkopf [26]. Instead of the hyperplane, HSSVM uses a hypersphere for pattern recognition. HSSVM can not only separate two different classes, but also divide the sample space into two different parts [27]. Moreover, in order to overcome the limitations of inherent binary classification properties, hypersphere multiclass SVM (HMSVM) was introduced [28]. In HMSVM classification, the samples in same class are assigned to a hypersphere, therefore, the data space is composed of several hyperspheres [29]. Using HMSVM, the multi-class classification is realized directly. The quadratic programming calculation of HMSVM is less than that of one-class SVM, which causes shorter training and testing time. In this paper, particle swarm optimization (PSO) [30] is employed for parameter selection in HMSVM. Then the optimized classification model is applied to PD fault pattern recognition, using extracted PD features.

In this work, the proposed PD fault diagnosis method is combined with the excellent properties of both VMD and MDE. The characteristic parameters representing dominant PD features are effectively extracted. Besides, it can solve the problems in traditional PD feature extraction methods, such as the limitations of high request of sampling rate, slow speed of data processing, difficulties to extract PD phase information, influences by electromagnetic interference, difficulty of selection of wavelet basis, and so on. Finally, HMSVM is employed for PD pattern recognition with extracted parameters. To verify the effectiveness and superiority of the proposed method, different PD feature extraction methods and diverse classifiers are introduced. Results verify the exactness of the conclusion.

The rest of this paper is organized as follows: Section 2 describes the theories of VMD, MDE and HMSVM, and presents the PD fault diagnosis procedure. Section 3 presents a brief introduction to the experimental setup used to generate PD signals. In Section 4 we show the results with their validation. The paper ends with conclusions in Section 5.

## 2. PD Fault Diagnosis Based on VMD-MDE and HMSVM

### 2.1. VMD Algorithm

VMD decomposes one real signal into *K* independent sub-signal *u*_k_, which has specific sparsity. This procedure gets the minimum bandwidth estimation of each modal [31]. The procedure of signal decomposition is to solve the variational problem. The variational model with constraint condition is as follows:

(1) $$\left\{ \begin{array}{l} \underset{\left\{u_{k}\},\{w_{k}\right\}}{\min}\;\{\sum_{k}{\left\|\partial_{t}[(\delta (t)+\frac{j}{\pi t})u_{k}(t)]e^{-jw_{k}t}\right\|}_{2}^{2}\}\\ s.t.\;\sum_{k}u_{k}=f \end{array}\right.$$

where $\left\{u_{k}\}=\{u_{1},u_{2},\cdots ,u_{K}\right\}$ demonstrates the modal components, $\left\{w_{k}\}=\{w_{1},w_{2},\cdots ,w_{K}\right\}$ is the center frequency of each modal component, *δ*(*t*) represents impulse function, ∂*_t_* means the partial derivatives of *t*, and *f* is the original signal.

In order to obtain the optimal solution of such constrained variational problem, Lagrangian multiplier *λ*(*t*) is introduced. The constrained variational problem is transformed into non-constrained problem:

(2) $$L(\{u_{k}\},\{\omega_{k}\},\lambda )=\alpha \sum_{k}{\left\|\partial_{t}[(\delta (t)+\frac{j}{\pi t})u_{k}(t)]e^{-jw_{k}t}\right\|}_{2}^{2}+{\left\|f(t)-\sum_{k}u_{k}(t)\right\|}_{2}^{2}+\langle \lambda (t),f(t)-\sum_{k}u_{k}(t)\rangle$$

where *α* is the quadratic penalty factor. Alternate direction method of multipliers (ADMM) is introduced to obtain the saddle point of such Lagrangian function, which is the optimal solution. 

The procedure of VMD can be summarized in the following steps:

(1) Initialize each modal component $\left\{u_{k}^{1}\right\}$, center frequency $\left\{\omega_{k}^{1}\right\}$ and operators $\left\{\lambda^{1}\right\}$. Set *n* = 0.
(2) Update $u_{k}$ in non-negative frequency intervals:
  (3) $${\hat{u}}_{k}^{n+1}(\omega )\leftarrow \frac{\hat{f}(\omega )-\sum_{i<k}{\hat{u}}_{i}^{n+1}(\omega )-\sum_{i>k}{\hat{u}}_{i}^{n}(\omega )+\frac{{\hat{\lambda }}^{n}(\omega )}{2}}{1+2\alpha {\left(\omega -\omega_{k}^{n}\right)}^{2}}$$
(3) Update $\omega_{k}$.
  (4) $$\omega_{k}^{n+1}\leftarrow \frac{\int_{0}^{\infty }\omega {\left|{\hat{u}}_{k}^{n+1}(\omega )\right|}^{2}d\omega }{\int_{0}^{\infty }{\left|{\hat{u}}_{k}^{n+1}(\omega )\right|}^{2}d\omega }$$
(4) Update *λ* in non-negative frequency intervals:
  (5) $${\hat{\lambda }}^{n+1}\leftarrow {\hat{\lambda }}^{n}+\tau (\hat{f}(\omega )-\sum_{k}{\hat{u}}_{i}^{n+1}(\omega ))$$
(5) For a given precision ε>0, if $\frac{\sum_{k}{\left\|{\hat{u}}_{k}^{n+1}-{\hat{u}}_{k}^{n}\right\|}_{2}^{2}}{{\left\|{\hat{u}}_{k}^{n}\right\|}_{2}^{2}}<\varepsilon$, then stop iteration. Otherwise, return to (2).

### 2.2. Theory of Multiscale Dispersion Entropy

#### 2.2.1. Dispersion Entropy

For a univariate signal $x=x_{1},x_{2},\cdots ,x_{N}$, dispersion entropy method can be described in following steps [32]:

(1) Map $x_{j}(j=1,2,\cdots ,N)$ into $y=\{y_{1},y_{2},\cdots ,y_{N}\}$ from 0 to 1 with the normal cumulative distribution function:
  (6) $$y_{j}=\frac{1}{\sigma \sqrt{2\pi }}\int_{-\infty }^{x_{j}}e^{\frac{-{\left(t-\mu \right)}^{2}}{2\sigma^{2}}}dt$$
  where σ and μ represent the standard deviation and mean of *x*, respectively.
(2) Assign each *y*_j_ to an integer from Label 1 to *c* using a linear algorithm. The mapped signal can be defined as follows:
  (7) $$z_{j}^{c}=round(c.y_{j}+0.5)$$
(3) Define embedding vector $z_{i}^{m,c}$ with embedding dimension *m* and time delay *d* as:
  (8) $$z_{i}^{m,c}=\{z_{i}^{c},z_{i+d}^{c},\cdots ,z_{i+(m-1)d}^{c}\},\;i=1,2,\cdots ,N-(m-1)d$$
  Each time series $z_{i}^{m,c}$ is mapped to a dispersion pattern $\pi_{v_{0}v_{1}\cdots v_{m-1}}$, where:
  $$z_{i}^{c}=v_{0},z_{i+d}^{c}=v_{1}.\cdots ,z_{i+(m-1)d}^{c}=v_{m-1}$$
(4) For each dispersion pattern, the relative frequency can be obtained as:
  (9) $$p(\pi_{v_{0}v_{1}\cdots v_{m-1}})=\frac{Number\{i|i\leq N-(m-1)d,z_{i}^{m,c}\;has\;\mathrm{type}\;\pi_{v_{0}v_{1}\cdots v_{m-1}}\}}{N-(m-1)d}$$
  where $p(\pi_{v_{0}v_{1}\cdots v_{m-1}})$ represent the number of dispersion pattern $\pi_{v_{0}v_{1}\cdots v_{m-1}}$, which is assigned to $z_{i}^{m,c}$ divided by the total number of embedding signals with embedding dimension *m*.
(5) Based on Shannon’s definition of entropy, dispersion entropy with embedding dimension *m*, time delay *d*, and the number of classes *c* can be defined as
  (10) $$DE(x,m,c,d)=-\sum_{\pi =1}^{c^{m}}p(\pi_{v_{0}v_{1}\cdots v_{m-1}})\cdot \ln(p(\pi_{v_{0}v_{1}\cdots v_{m-1}}))$$

#### 2.2.2. Multiscale Dispersion Entropy

Multiscale Dispersion Entropy (MDE) is the combination of the coarse-graining with dispersion entropy. In MDE, the original signal $x=x_{1},x_{2},\cdots ,x_{N}$ of length *N* is first divided into non-overlapping scale factor *τ*. Then the new coarse-grained signals can be shown as follows:

(11) $$x_{j}^{\left(\tau \right)}=\frac{1}{\tau }\sum_{i=(j-1)\tau +1}^{j\tau }x_{i},\;1\leq j\leq N/\tau$$

Calculate the entropy value of each coarse-grained signal of length *N*/τ with dispersion entropy method:

(12) $$MDE(x,\tau ,m,c,d)=DE(x^{\left(\tau \right)},m,c,d)$$

### 2.3. Theory of HMSVM

#### 2.3.1. HMSVM

**Figure 1:**
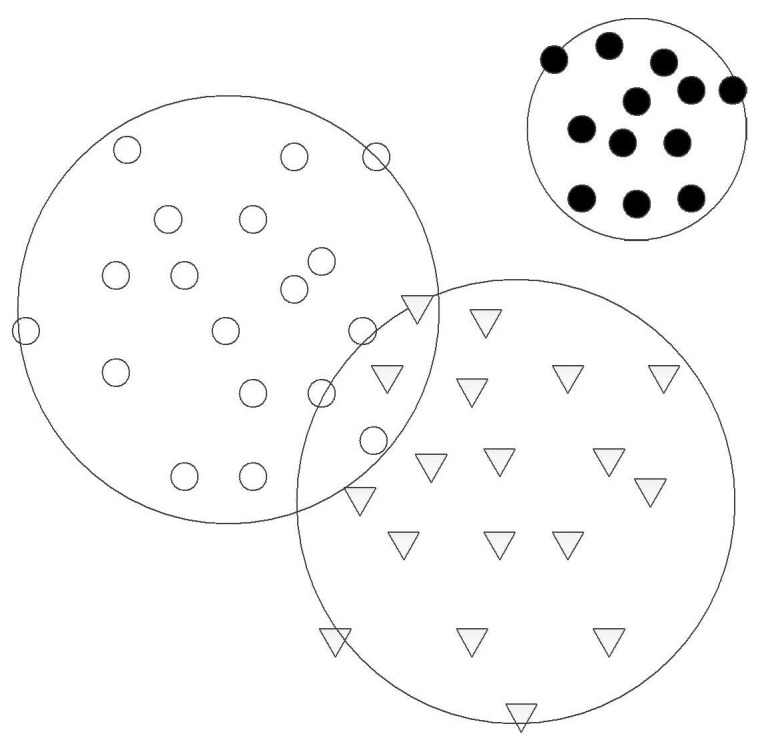
Classification model of HMSVM.

HMSVM can classify the samples directly. Each type of samples needs only one-hypersphere training. All training samples are mapped into high-dimension space. Each type of training samples searches for one hypersphere that has small radius and more target samples. HMSVM classification model is shown in Figure 1.

For an *M*-class problem, a collection of elements *X_m_* (*m* = 1, 2, …, *M*) is given. Assume that each *X_m_* contains *m*-dimension sample *x_mi_*, *i* = 1, 2…*l_m_*, which represents *i*-th element in *m*-class.

Assign one hypersphere (*a_m_*,*R_m_*) for each sample *X_m_*, where *a_m_* is the center of sphere, *R_m_* is the radius of suprasphere. The objective function of *m*-th suprasphere can be defined as follows:

(13) $$\begin{array}{l} \underset{R_{m}}{\min}(R_{m}^{2}+C_{m}\sum_{i=1}^{l_{m}}\xi_{m,i})\\ s.t.\|\Phi \left(x_{m,i}\right)-a_{m}\|\leq R_{m}^{2}+\xi_{m,i},\xi_{m,i}\geq 0 \end{array}$$

where *C*_m_ is the penalty factor, representing the trade-off between *R_m_* and target samples. *ξ_m_*_,*i*_ is the slack variable of HMSVM allowing remote samples staying outside the sphere.

Lagrange function can be obtained after Lagrange multiplier is introduced:

(14) $$L(R,a,\xi ,\alpha ,\gamma )=R_{m}{}^{2}+C_{m}\sum_{i=1}^{l_{m}}\xi_{mi}-\sum_{i=1}^{l_{m}}\alpha_{i}\{R^{2}+\xi_{mi}-(\left\|x^{2}\right\|-2a\cdot x_{i}+\left\|a^{2}\right\|)\}-\sum_{i=1}^{l_{m}}\gamma_{i}\xi_{mi}$$

The derivative operation of Equation (14) is processed to obtain the dual optimization problem as follows:

(15) $$\underset{a_{m}}{\min}\sum_{i}\sum_{j}\alpha_{m}{}_{,i}\alpha_{m,j}K(x_{m,i},x_{m,j})-\sum_{i=1}^{l_{m}}\alpha_{m,j}K(x_{m,i},x_{m,j})$$

The restricting condition that the target function should satisfy is shown as follows:

(16) $$\sum_{i=1}^{l_{m}}\alpha_{m,i}=1,\;0\leq \alpha_{m,i}\leq C_{m}$$

For an unknown fault sample *d*, we first calculate the square of the distance between *d* and *a_m_* using the formula below:

(17) $$D^{2}(d)={\left\|d-a_{m}\right\|}^{2}=(d\cdot d)-2\sum_{i=1}^{l_{m}}\alpha_{i}(d\cdot x_{i})+\sum_{i=1}^{l_{m}}\sum_{j=1}^{l_{m}}\alpha_{i}\alpha_{j}(x_{i}\cdot x_{j})$$

The radius of the suprasphere is defined as *R_m_ = D*(*x*_i_), where *x*_i_ represents the support vector. Therefore, the category assigned to the unknown sample *d* can be determined according to the comparison between *R*_m_ and *D*(*d*).

#### 2.3.2. Kernel Function Selection

Due to the complexity among different PD fault samples, the spherical distribution will not appear in low-dimensional space. PD fault samples need to be mapped into high-dimension space using kernel functions to obtain the optimal hypersphere. In recent time, the common kernel functions include radial basic function (RBF) [33], polynomial kernel function and sigmoid function. After repeating tests, RBF shows outstanding performance. Therefore, RBF is selected as the kernel function for HMSVM. It can be defined in Equation (18):

(18) $$K(x,x_{i})=\exp\{-\frac{{\left|x-x_{i}\right|}^{2}}{\sigma^{2}}\}$$

### 2.4. PD Fault Diagnosis Based on VMD-MDE and HMSVM

In this paper, the proposed PD fault diagnosis method combines feature extraction and pattern recognition. Firstly, the original PD signal is decomposed using VMD to obtain the intrinsic mode functions. Secondly MDE value of each intrinsic mode function is calculated. And then principal component analysis (PCA) [34] is introduced to select principal components of MDE as PD feature vectors. Finally, the extracted vectors are sent to HMSVM pattern classifier to recognize different PD faults. The fault diagnosis procedure is as follows:

Step 1: Extract different types of PD signals in experimental environment, including floating discharge (FD), needle-surface discharge (ND), ball-surface discharge (BD) and corona discharge (CD).

Step 2: Select proper initial number of IMF according to the center frequency observation and decompose PD signals using VMD into intrinsic mode functions with different characteristic scales.

Step 3: Calculate the correlation coefficients between each IMF and original PD signal to select effective IMFs [35,36]. If the coefficient is greater than the threshold value, then keep the IMF as effective one. Otherwise, abandon the IMF. In this paper, the threshold value of the correlation coefficient is set to 0.3.

Step 4: Fix the decomposition scale for IMF and calculate the MDE value of extracted IMFs as original PD feature vectors.

Step 5: Analyze the PD vectors by PCA and extract fewer representative principal components as PD characteristic parameters.

Step 6: Send extracted PD characteristic parameters into HMSVM classifier to diagnose different PD fault modes and obtain the final diagnosis result.

**Figure 2:**
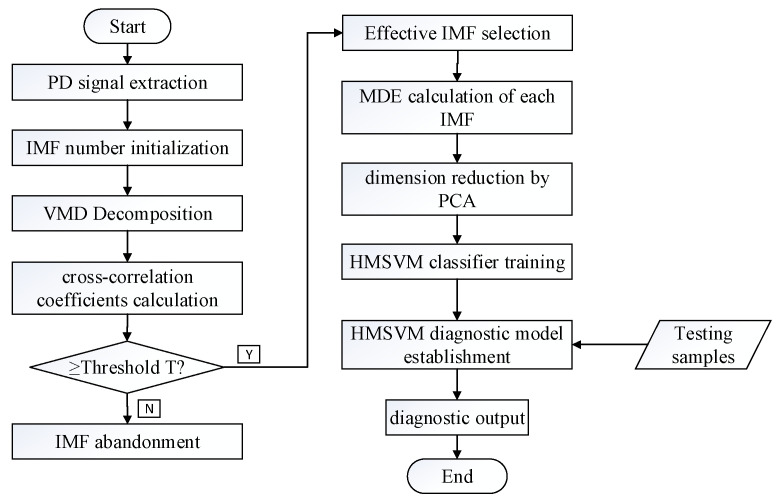
PD fault diagnosis procedure based on VMD-MDE and HMSVM.

The flow chart of PD fault diagnosis with proposed method is shown in Figure 2.

## 3. Experiments and Analysis

### 3.1. Experimental Setup

**Figure 3:**
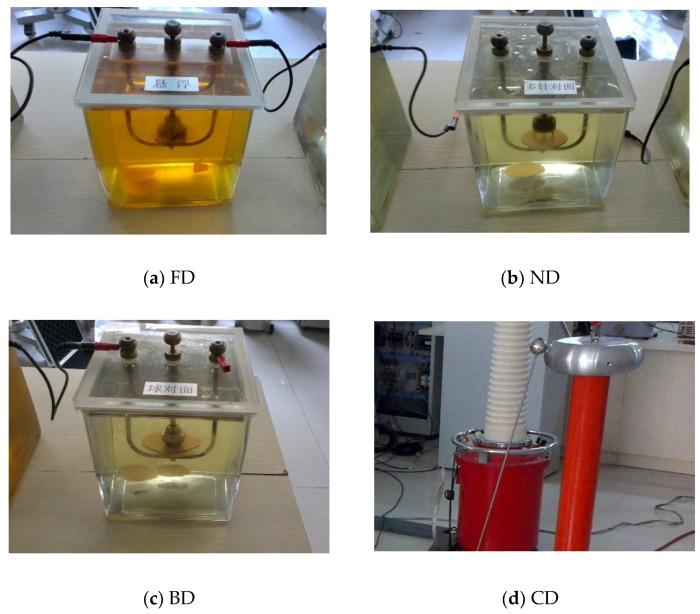
PD models.

**Figure 4:**
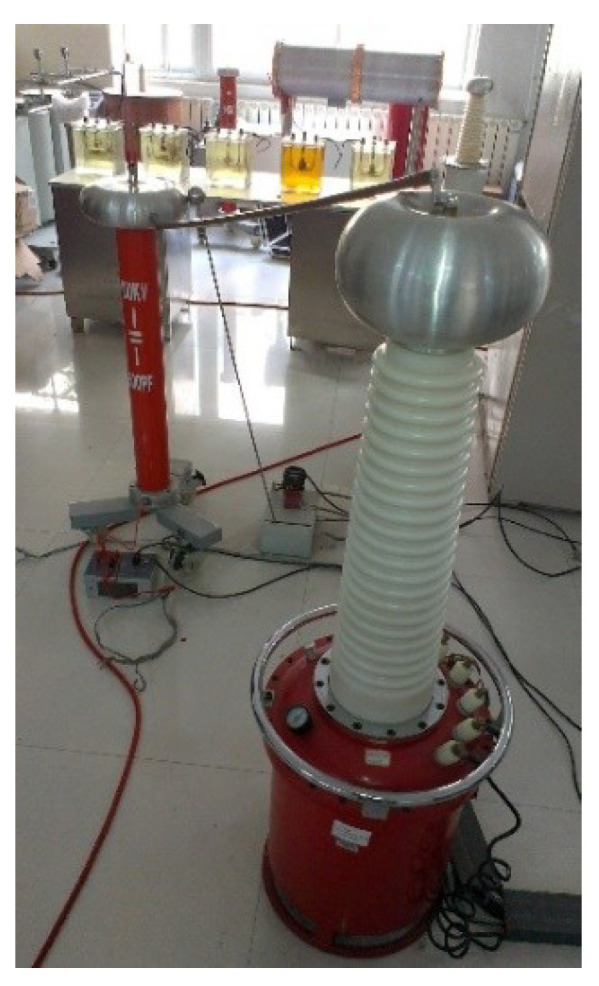
Photograph of experimental setup.

Different PD types can produce different effects in insulation materials, but the range may be diverse. To analyze the characteristics of different PD types, PD signals of different models are extracted in the laboratory [37]. According to the inner insulation structure of power transformers, there are four possible different PD types, including FD, ND, BD and CD. PD models are shown in Figure 3. The experimental setup is shown in Figure 4.

**Figure 5:**
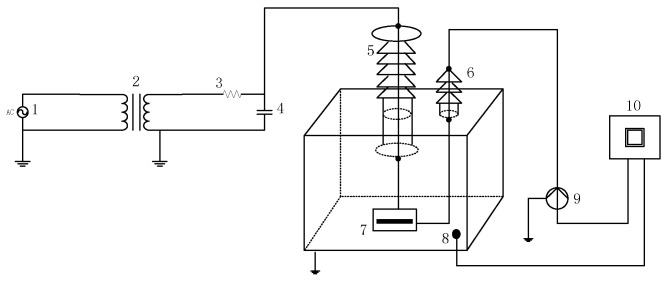
The connection diagram of PD experiment. 1—AC power source; 2—step up transformer; 3—resistance; 4—capacitor; 5—high voltage bushing; 6—small bushing; 7—PD model; 8—UHF sensor; 9—current sensor; 10—console.

PD signals are detected in the simulated transformer tank in the laboratory. The pulse current is collected by a current sensor with a 500 kHz–16 MHz bandwidth. The UHF signal is extracted by a UHF sensor with a 10–1000 MHz bandwidth. The signal received is imported into the PD analyzer. The test condition is shown in Table 1 and the experimental connection diagram is shown in Figure 5.

### 3.2. Signal Extraction

**Figure 6:**
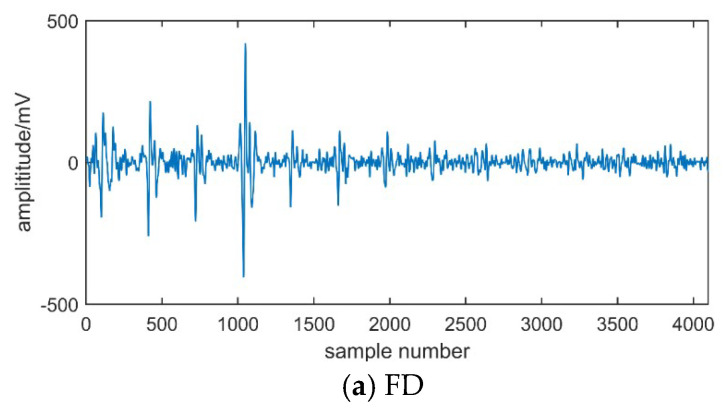

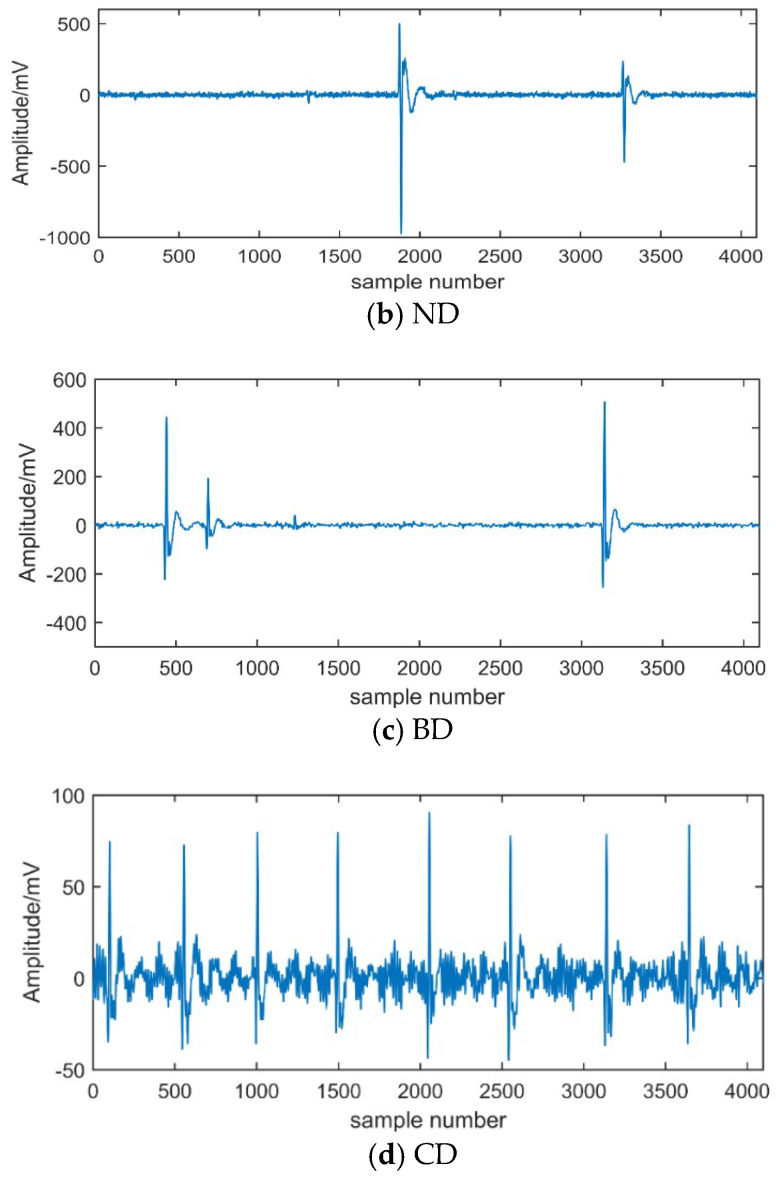
PD signals.

In this paper, four different types of PD signals are extracted with above experimental setup. The extracted PD waveforms are shown in Figure 6.

## 4. Results and Analysis

### 4.1. VMD Decomposition

In this paper, float discharge is taken as an example for VMD decomposition. The number of IMFs, represented as *K*, is determined according to the central frequency observation. The central frequency of IMF with the variation of *K* is shown in Table 2.

**Figure 7:**
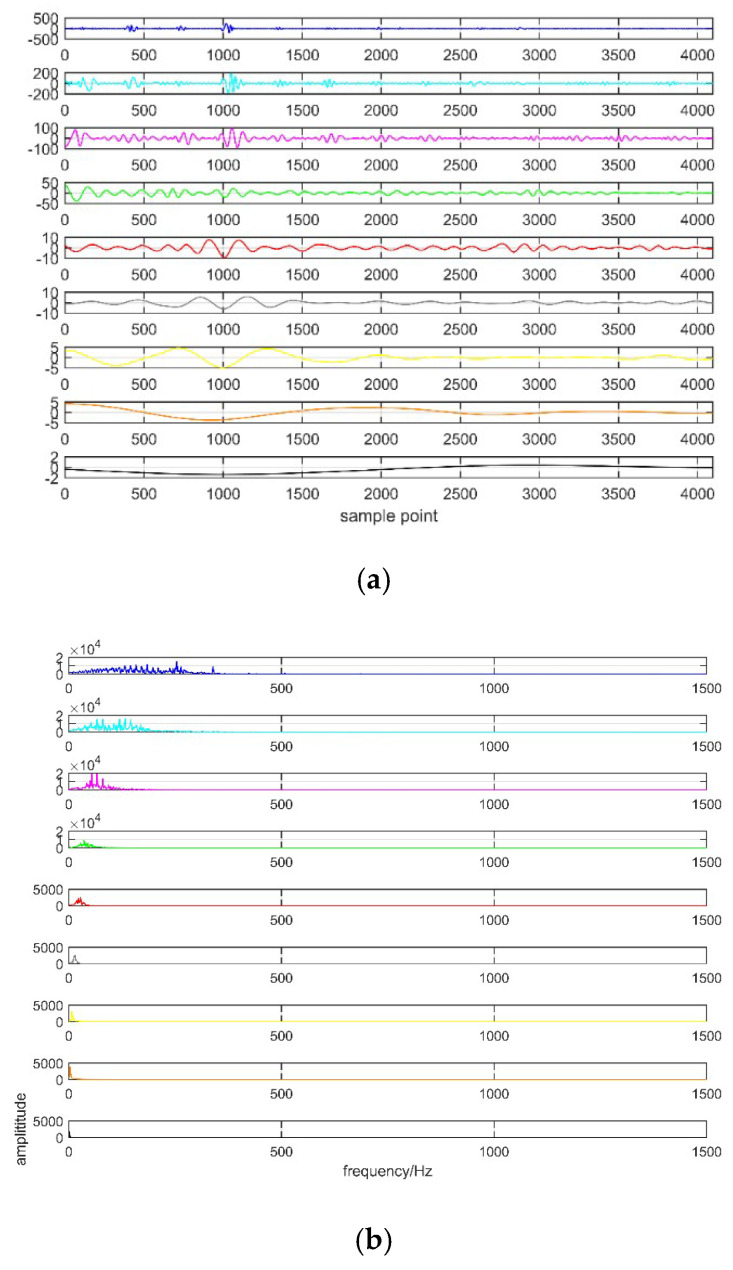
Results of EMD decomposition. (**a**) IMF of decomposition; (**b**) Frequency spectrum of decomposition.

**Figure 8:**
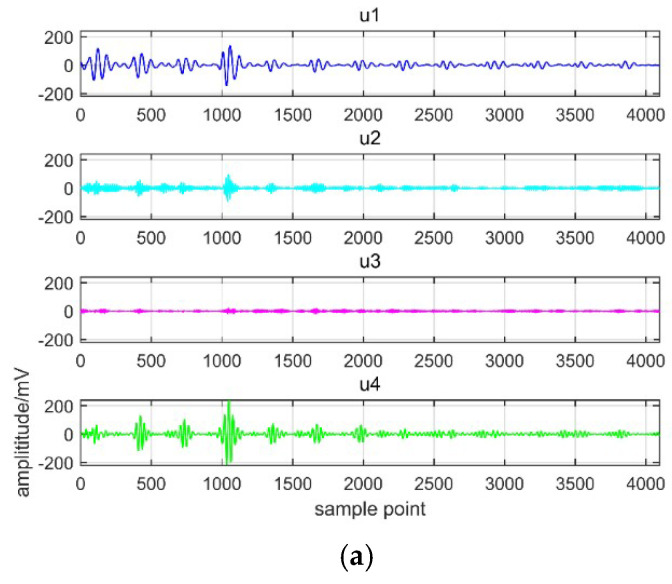

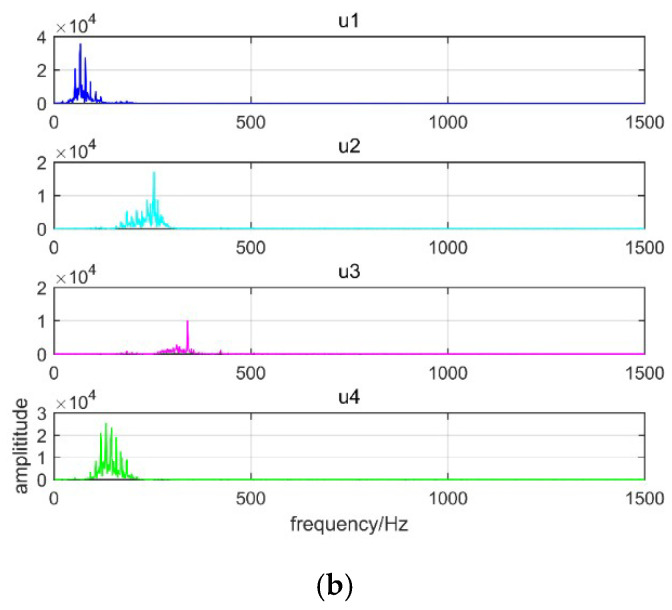
Results of VMD decomposition. (**a**) IMF of decomposition; (**b**) Frequency spectrum of decomposition.

Table 2 shows that the IMFs with similar central frequency arise from *K* = 5, which means excessive decomposition. Therefore *K* = 4 is selected as the number of IMF. In this paper, the balancing parameter *α* = 2000 and bandwidth parameter *τ* = 0.1. The decomposition results with EMD and VMD are shown in Figure 7 and Figure 8.

Figure 7 shows the EMD decomposition results containing IMF components and frequency spectrum. From the figure we can see that eight IMF components and one remaining component are obtained. However, the problem of mode mixing occurs in EMD decomposition. Besides, IMF component in each decomposition level is different from that of original signal. Figure 8 describes the results of VMD decomposition. It can be seen from this figure that the modal components in VMD approach to the real signal. Figure 7 and Figure 8 verify the effectiveness of VMD and the superiority over EMD. It can be concluded that VMD is more suitable for PD signal decomposition.

### 4.2. IMF Selection

In order to obtain the effective IMF, the correlation coefficient (CC) between each IMF and original PD signal is calculated. Given a threshold *T*, if the CC is greater than *T*, the IMF will be selected as effective component; otherwise it will be regarded as false component and abandoned. In this work, *T* is set to 0.3. The CC values of IMF for VMD and EMD are shown as Table 3.

Table 3 shows that the CC value of first three IMFs is larger than the given threshold, which means these IMFs could represent the real components of PD signals. Therefore, the first three IMFs are selected and analyzed for VMD decomposition. Similarly, we can see that the CC value is smaller than the threshold from the fourth IMF, which means these IMFs contain less information of PD signals. Consequently, the first four IMFs are kept for EMD decomposition.

### 4.3. Feature Extraction

In this paper four different types of PD signals are decomposed using VMD method. The VMD decomposition parameters are shown in Table 4. *K*_s_ is the number of effective IMFs calculated as described in Section 4.2.

**Figure 9:**
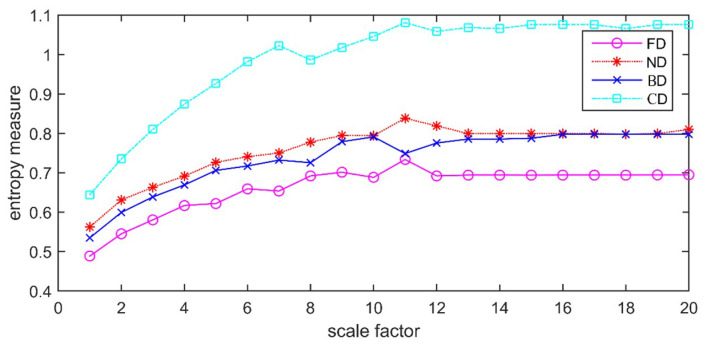
MDE variation with scale factors.

Using the above parameters, the corresponding IMFs of different types of PD are obtained by VMD decomposition. Then the MDE value of each IMF is calculated. During MDE calculation, some preset parameters need to be given, including scale factor *s*, number of classification *c*, time delay *d* and embedded dimension *m*. But considering that aliasing may occur when *d* > 1, *d* is set to 1 as recommended. In order to avoid the trivial case of only one dispersion pattern, *c* is set to 2. For better detection on dynamic change of signals, *m* is set to 6. To analyze the variation of MDE values with different scales, *s* is set to 20. With above parameters, MDE values of four different types of PD signals extracted in the laboratory are calculated. For each type of PD, MDE values are averaged with different IMFs, shown in Figure 9.

**Figure 10:**
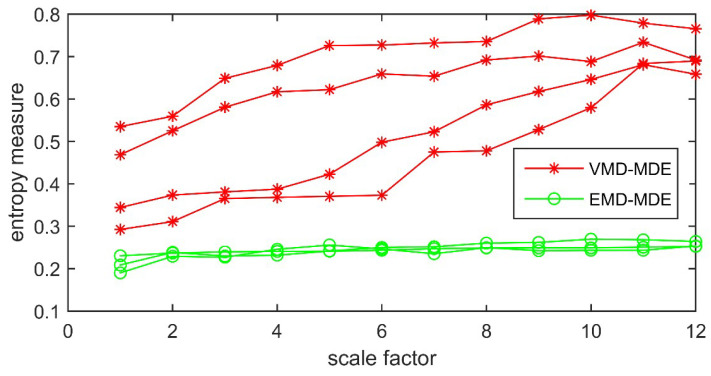
MDE values of IMFs using VMD and EMD.

Figure 9 shows that different types of PD signals have diverse MDE values with variations of scale factors. The reason is that the randomness of PD signals is changing when PD fault occurs, which could change the MDE values. It also indicates that a single scale cannot completely reflect all the signal information and much more important information distributes in other scales. MDE can effectively detect the dynamic variation of PD signals which represent the fault characteristics with different scales. It can be found from the figure that MDE values start to level off after Scale 12. Therefore, the scale factor is set to 12 in this paper. In the case of FD, MDE values of IMFs using VMD and EMD are shown in Figure 10.

Figure 10 shows that with the variation of scales, MDE values extracted by VMD are different. However, MDE values extracted by EMD seems to be same with the increase of decomposition scales which makes it difficult to distinguish different IMFs. The initial FD feature vectors combined with the MDE of all IMFs using VMD decomposition are shown in Table 5.

### 4.4. PCA-Based Dimension Reduction

Due to the high dimension of extracted feature vectors, it will cause big burden for pattern classifiers which can directly affect the recognition accuracy. In this paper, the PCA method is employed for dimension reduction of initial feature vectors. In the case of *K*_1_, the covariance matrix is constructed to obtain the principal components. The eigenvalue and eigenvector of the covariance matrix are solved for linear transformation of original vectors. To achieve the goal of dimension reduction, those factors whose eigenvalues are greater than 1 are selected as principal components. The eigenvalue and corresponding contribution rates of the covariance matrix are shown in Table 6.

**Figure 11:**
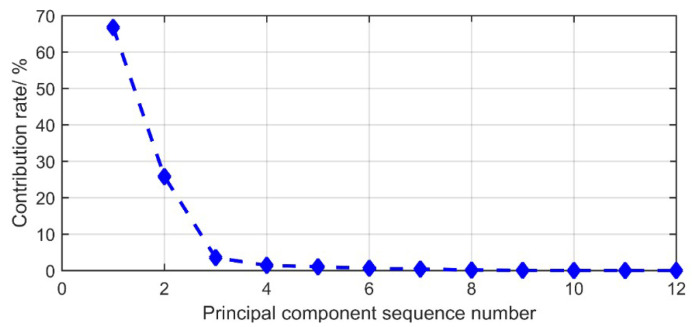
The variation of contribution rate with principle components.

Table 6 shows that first two eigenvalues are greater than 1, and the accumulated contribution rate is larger than 90%. The contribution rate changes with the variation of principle components, shown in Figure 11.

It can be concluded from above figure that, the contribution rate from the third principle component starts to level off. In addition, the contribution rates are decreasing gradually which can be ignored. Therefore, first two principle components are suitable for further analysis which represent most of the vector information. To do so, the original 12 indicators are reduced to 2 new ones. With a similar method, the principle components of *K*_2_, *K*_3_ and *K*_4_ can be obtained, shown in Table 7.

It can be seen from Table 7 that nine principle components factors are extracted from 48 feature vectors. And the contribution rate in each IMF is greater than 80%. Given the above, the dimension of feature vectors is reduced to nine after dimension reduction using PCA. Similarly, with above procedure, the calculated PD parameters of different PD types are shown in Table 8.

### 4.5. PD Pattern Recognition

In this paper, 400 PD samples, including FD, ND, BD and CD, are extracted in the laboratory containing 100 samples in each PD type. MDE values of four different PD types are calculated and 50 samples in each type constitute the initial feature vectors. To verify the effectiveness and superiority of the proposed method, the feature extraction methods based on multi-scale sample entropy (MSE) and multi-scale permutation entropy (MPE) are introduced. The calculation method of MSE and MPE is similar with that of MDE. Firstly, PD signals are decomposed using EMD or VMD. After that MSE or MPE values of extracted IMFs are calculated. Finally, PCA is applied to dimension reduction. The parameters during signal decomposition are shown in Table 9.

PD feature vectors extracted with the above three methods are sent to the HMSVM classifier. Due to the big impact on the fault diagnosis result, HMSVM parameters need optimal configuration with PSO. In the case of VMD-MDE method, first of all, PD samples are divided into training and testing samples. After multiple experimental trials, the number of particle population is set to 20, *w* = 1, *c*_1_ = 2, *c*_2_ = 2, the maximum number of iterations *N* = 200. The penalty parameter *C* is between 1/n and 1, while the searching range of the kernel parameter *σ* is between 1 and 100. The optimum fitness reaches the maximum value of 96.98% after 19 iterations, when *σ* = 12.26 and *C* = 0.35. Similarly, HMSVM parameters with different feature extraction methods are obtained as follows.

**Figure 12:**
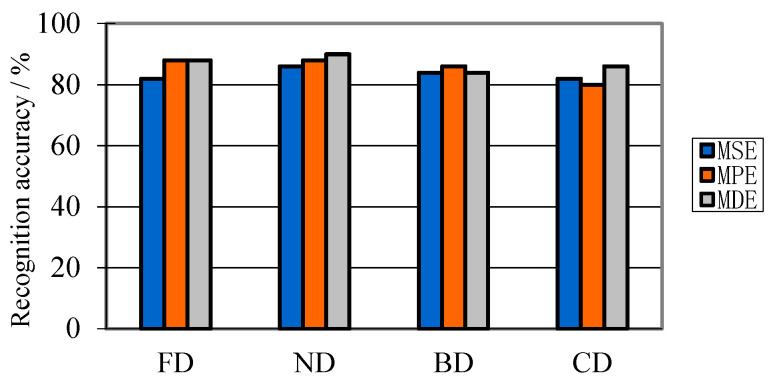
Recognition results using EMD decomposition.

**Figure 13:**
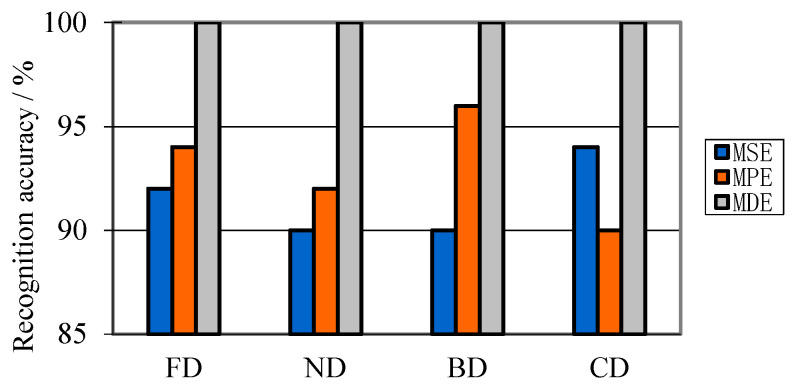
Recognition results using VMD decomposition.

Using the parameters in Table 10, HMSVM classifier is constructed for fault diagnosis of three different PD features. The recognition results with EMD and VMD decomposition are shown in Figure 12 and Figure 13.

Figure 12 and Figure 13 demonstrate that the recognition result using EMD decomposition is significantly different with that using VMD decomposition. Figure 12 illustrates that the recognition accuracy in each PD type is not less than 80% but no more than 90%, which means, using EMD decomposition, extracted PD features cannot represent most of signal characteristics. In contrary, Figure 13 shows that the recognition accuracy in each PD type is no less than 90%. Moreover, in each PD type, there’s no misjudged sample with MDE. This means that, with VMD decomposition, PD features can effectively represent most of signal information. Besides, from above two figures, it gets a satisfactory result with MDE parameters.

To compare the diagnosis results of PD features with different classifiers, artificial neural network (ANN) [38] and support vector machine (SVM) classifiers are introduced for PD pattern recognition. In ANN, back-propagation network is employed as the recognition model, which trains the weight with differentiable nonlinear functions. The classifier parameters are shown in Table 11. *σ* is the kernel parameter of RBF and *C* is the penalty factor in SVM.

**Figure 14:**
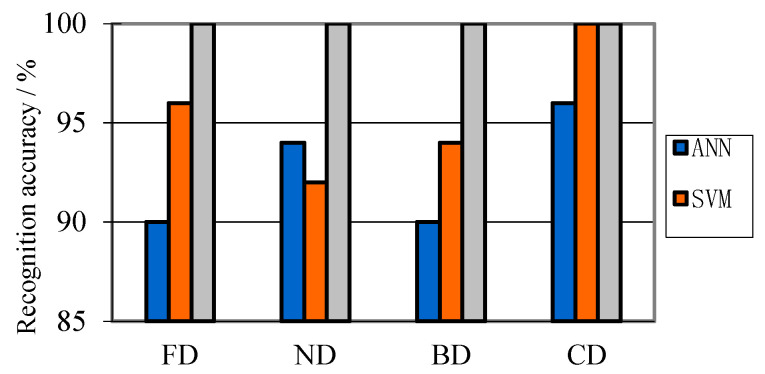
Recognition results using VMD-MDE method.

With the parameters shown in Table 10 and Table 11, ANN, SVM and HMSVM classifiers are constructed for PD pattern recognition. Using diverse classifiers, the recognition result with VMD-MDE can be seen in Figure 14. Table 12 shows the integrative result using different PD features, in which running time means the time used for PD fault diagnosis.

As can be illustrated in Figure 14, using the same PD feature extraction method, the recognition results with different classifiers are significantly different. The average classification accuracy achieved using HMSVM is 100.00%. HMSVM shows great advantages over ANN and SVM. Table 12 shows diverse diagnostic results with different PD features. Compared with different PD feature types, VMD-MDE gives less running time and higher recognition accuracy. It means parameters using VMD-MDE can represent most of PD signal components. The quadratic programming calculation of HMSVM is less than that of SVM, which causes shorter training and testing time. In addition, HMSVM shows better classification ability than other two classifiers, ANN and SVM.

## 5. Conclusions

In this paper, a novel PD fault diagnosis method is proposed. This method combines PD feature extraction based on VMD-MDE and PD pattern recognition based on HMSVM. First of all, four types of PD signals are extracted in the experimental environment, including FD, ND, BD and CD. Then VMD is employed for PD signal decomposition. Secondly, proper IMFs are selected according to central frequency observation and MDE values in each IMF are calculated. Afterwards PCA is introduced to select effective principle components in MDE as final PD characteristic parameters. Finally, the extracted principle factors are used as PD features and sent to the HMSVM classifier. Experiment results show the following advantages: the proposed method can extract effective IMFs according to VMD decomposition. PD feature information in IMFs can be quantified successfully with MDE. Using PCA, the principle components which represent prominent characteristics are effectively selected. With small data size and low computational complexity, this approach overcomes the limitations in traditional PD feature extraction methods. Compared with PD feature extraction methods based on EMD-MSE, EMD-MPE, EMD-MDE, VMD-MSE and VMD-MPE, this proposed approach based on VMD-MDE achieves higher recognition accuracy and needs less running time, which can improve the diagnosis efficiency to satisfy real time requirements.

HMSVM uses one hypersphere for pattern recognition. HMSVM can not only separate two different classes, but also divide the sample space into two different parts. Using HMSVM, the classification of multi-classes was realized directly. Compared with ANN and SVM classifiers, HMSVM obtains higher recognition rate and improves the accuracy and efficiency in PD fault diagnosis. On the whole, this proposed method provided a new scheme for PD fault diagnosis. For further consideration, the proposed fault diagnosis method can be employed in PD on-line monitoring and diagnosis.

## Figures and Tables

**Table 1:** Test condition of PD models.

PD Types	Initial Voltage/kV	Breakdown Voltage/kV	Testing Voltage/kV	Sample Number
FD	2	7	3/4	50/50
ND	8.8	12	9/10	50/50
BD	3.5	10	5/6	50/50
CD	4.5	10	6/7	50/50

**Table 2:** Central frequency.

Number of IMFs	Central Frequency/MHz						
2	0.0079	7.3682					
3	0.0073	6.9573	12.3268				
4	0.0059	6. 8232	11.9803	13.2581			
5	0.0055	6. 8041	12.0256	13.1263	13.3572		
6	0.0059	6. 7855	11.7785	13.5579	13.2602	13.9348	
7	0.0053	6. 8034	12.1379	13.7877	13.9021	13.9975	14.2814

**Table 3:** CC values.

	*u*1	*u*2	*u*3	*u*4	*u*5	*u*6	*u*7	*u*8	*u*9
VMD	0.6809	0.5129	0.3583	0.0083	-	-	-	-	-
EMD	0.7362	0.6035	0.4231	0.3026	0.2092	0.1123	0.0365	0.0086	0.0025

**Table 4:** VMD decomposition parameters.

PD Type	*K*	*α*	*τ*	*K*_s_
FD	4	2000	0.1	3
ND	5	2000	0.1	3
BD	4	2000	0.1	4
CD	4	2000	0.1	4

**Table 5:** Initial feature vectors.

IMF	Vectors
*K*_1_	*O*_1_, *O*_2_, *O*_3_, *O*_4_, *O*_5_, *O*_6_, *O*_7_, *O*_8_, *O*_9_, *O*_10_, *O*_11_, *O*_12_
*K*_2_	*P*_1_, *P*_2_, *P*_3_, *P*_4_, *P*_5_, *P*_6_, *P*_7_, *P*_8_, *P*_9_, *P*_10_, *P*_11_, *P*_12_
*K*_3_	*Q*_1_, *Q*_2_, *Q*_3_, *Q*_4_, *Q*_5_, *Q*_6_, *Q*_7_, *Q*_8_, *Q*_9_, *Q*_10_, *Q*_11_, *Q*_12_
*K*_4_	*R*_1_, *R*_2_, *R*_3_, *R*_4_, *R*_5_, *R*_6_, *R*_7_, *R*_8_, *R*_9_, *R*_10_, *R*_11_, *R*_12_

**Table 6:** Eigenvalues and corresponding contribution rates.

Vectors	Eigenvalue	Contribution Rate/%	Accumulated Contribution Rate/%
*O*_1_	3.732	66.738	66.738
*O*_2_	2.169	25.843	92.581
*O*_3_	0.852	3.560	96.141
*O*_4_	0.603	1.435	97.576
*O*_5_	0.304	1.064	98.64
*O*_6_	0.124	0.626	99.266
*O*_7_	0.102	0.441	99.707
*O*_8_	0.075	0.152	99.859
*O*_9_	0.052	0.086	99.945
*O*_10_	0.036	0.027	99.972
*O*_11_	0.029	0.024	99.996
*O*_12_	0.003	0.004	100.00

**Table 7:** Principle components with different IMFs.

IMF	KMO	Contribution Rate/%	Principle Component
*K*_1_	0.852	92.581	*O*_1_, *O*_2_
*K*_2_	0.767	88.379	*P*_1_, *P*_2_
*K*_3_	0.734	80.232	*Q*_1_, *Q*_2_, *Q*_3_
*K*_4_	0.752	83.368	*R*_1_, *R*_2_

**Table 8:** Principle components with different IMFs.

PD Type	Parameters				
	*K*_1_	*K*_2_	*K*_3_	*K*_4_	*K*_5_
FD	*O*_1_, *O*_2_	*P*_1_, *P*_2_	*Q*_1_, *Q*_2_, *Q*_3_	*R*_1_, *R*_2_	-
ND	*O*_1_, *O*_2_	*P*_1_, *P*_2_	*Q*_1_, *Q*_2_	*R*_1_, *R*_2_	*S*_1_, *S*_2_
BD	*O*_1_, *O*_2_, *O*_3_	*P*_1_, *P*_2_	*Q*_1_, *Q*_2_	*R*_1_, *R*_2_	-
CD	*O*_1_, *O*_2_	*P*_1_, *P*_2_, *P*_3_	*Q*_1_, *Q*_2_	*R*_1_, *R*_2_	-

**Table 9:** Parameters selection.

	EMD Decomposition			VMD Decomposition		
	Level	Scale	Principle Components Number	Level	Scale	Principle Components Number
MSE	4	14	10	3	12	8
MPE	3	10	8	3	10	8
MDE	3	12	9	4	12	9

**Table 10:** HMSVM parameters.

	EMD-MSE	EMD-MPE	EMD-MDE	VMD-MSE	VMD-MPE	VMD-MDE
*C*	0.43	0.31	0.27	0.46	0.33	0.35
σ	10.38	11.86	10.19	12.05	9.37	12.26

**Table 11:** Parameters of ANN and SVM.

Classifier	Type	EMD-MSE	EMD-MPE	EMD-MDE	VMD-MSE	VMD-MPE	VMD-MDE
SVM	*C*	0.25	0.28	0.45	0.44	0.38	0.46
	*σ*	8.39	10.57	8.32	9.18	8.25	10.22
ANN	Input	10	8	9	8	8	9
	Output	4	4	4	4	4	4
	Hidden layer	16	12	14	12	10	12

**Table 12:** Recognition result with different PD features.

Feature Types	ANN		SVM		HMSVM	
	Recognition Accuracy/%	Running Time/s	Recognition Accuracy/%	Running Time/s	Recognition Accuracy/%	Running Time/s
EMD- MSE	86.00	6.88 × 10^−4^	88.50	6.92 × 10^−4^	86.50	6.75 × 10^−4^
EMD- MPE	86.50	3.45 × 10^−3^	84.00	3.21 × 10^−3^	86.00	3.51 × 10^−3^
EMD- MDE	88.00	5.39 × 10^−4^	90.50	5.36 × 10^−4^	91.50	1.68 × 10^−3^
VMD- MSE	95.00	8.16 × 10^−4^	96.50	7.29 × 10^−4^	97.50	7.80 × 10^−4^
VMD- MPE	98.00	7.45 × 10^−4^	97.50	7.12 × 10^−4^	99.00	7.42 × 10^−4^
VMD- MDE	98.00	5.36 × 10^−4^	99.00	5.32 × 10^−4^	100.00	5.27 × 10^−4^
